# Mechanism of Anti-bacterial Activity of Zinc Oxide Nanoparticle Against Carbapenem-Resistant *Acinetobacter baumannii*

**DOI:** 10.3389/fmicb.2018.01218

**Published:** 2018-06-06

**Authors:** Vishvanath Tiwari, Neha Mishra, Keval Gadani, P. S. Solanki, N. A. Shah, Monalisa Tiwari

**Affiliations:** ^1^Department of Biochemistry, Central University of Rajasthan, Ajmer, India; ^2^Department of Physics, Saurashtra University, Rajkot, India

**Keywords:** *Acinetobacter baumannii*, microbial drug resistance, antimicrobial nanoparticles, mechanism of ZnO action, ROS

## Abstract

*Acinetobacter baumannii* is a multi-drug resistant opportunistic pathogen, which causes respiratory and urinary tract infections. Its prevalence increases gradually in the clinical setup. Carbapenems (beta-lactam) are most effective antibiotics till now against *A. baumannii*, but the development of resistance against it may lead to high mortality. Therefore, it is of utmost importance to develop an alternative drug against *A. baumannii*. In the present study, we have synthesized ZnO nanoparticle (ZnO-NP) and characterized by X-ray diffraction, Fourier transform infrared (FTIR) spectroscopy and UV-Visible spectroscopy. Prepared ZnO-NPs have the size of 30 nm and have different characteristics of ZnO-NPs. Growth kinetics and disk diffusion assay showed that ZnO-NP demonstrated good antibacterial activity against carbapenem resistant *A. baumannii*. We have also investigated the mechanism of action of ZnO-NPs on the carbapenem resistant strain of *A. baumannii*. The proposed mechanism of action of ZnO involves the production of reactive oxygen species, which elevates membrane lipid peroxidation that causes membrane leakage of reducing sugars, DNA, proteins, and reduces cell viability. These results demonstrate that ZnO-NP could be developed as alternative therapeutics against *A. baumannii*.

## Introduction

*Acinetobacter baumannii* is a Gram-negative, strictly aerobic, catalase-positive, non-motile, non-fermenting, non-fastidious coccobacilli (Peleg et al., 2008), and mainly found in the hospital setups (Fournier and Richet, 2006) but rarely on human skin (Seifert et al., 1997; Berlau et al., 1999) and fingertips (Glew et al., 1977). This pathogen targets hospitalized patients who are critically ill and cracks in the skin and respiratory tract (Peleg et al., 2008). It can grow across a varying range of temperatures, pHs, and nutrient levels, making pathogen highly adapted to survival in both human or environmental vectors (Choi et al., 2005). Carbapenems (beta-lactams) are prescribed by doctors against it. Different mechanisms of resistance against carbapenem have been explained for *A. baumannii*, such as alteration of outer membrane proteins (Vashist et al., 2010), altered penicillin-binding proteins (Vashist et al., 2011), acquire carbapenemases (Tiwari et al., 2012a,b; Tiwari and Moganty, 2013, 2014), efflux pumps (Verma et al., 2018), enhanced metabolism (Tiwari et al., 2012c; Tiwari, 2013), and biofilm formation (Tiwari et al., 2017a, 2018a; Roy et al., 2018). Therefore, there is an urgent need to design or develop an alternative drug to beta-lactams (carbapenem) that may be used to control *A. baumannii*. Different approaches have been investigated that includes screening of herbal compounds (Tiwari et al., 2015b, 2016), *in silico* drug designing (Tiwari et al., 2018a,b; Verma et al., 2018), nanomaterial-based approaches (Tiwari et al., 2015a, 2017b), etc. to find suitable alternative to the carbapenem.

The natural and synthetic polymers, metals, and metallic alloys offer several explicit properties that make them smart for biomedical applications (Fuku et al., 2016; Jesudoss et al., 2016; Kaviyarasu et al., 2017a,b; Maria Magdalane et al., 2017; Matinise et al., 2017). Among the nanoparticles, the metal oxide such as zinc oxide (ZnO) has got much attention in the recently because it is stable under diverse environmental conditions, and fabrication at low temperature (Dhage et al., 2005). ZnO particles shown antimicrobial activity (Raghunath and Perumal, 2017) against both Gram-positive (Guo et al., 2015), Gram-negative bacteria (Liu et al., 2009; Reddy et al., 2014; Guo et al., 2015), and even antibacterial activity against spores (Makhluf et al., 2005; Wagner et al., 2016). ZnO NPs are believed to be nontoxic, bio-safe, and biocompatible (Hameed et al., 2016). The anti-microbial activity of ZnO NPs has not studied on the carbapenem-resistant strain of *A. baumannii*. The mechanisms of antibacterial activity of ZnO particles are not well understood, although some statements were proposed such as, generation of hydrogen peroxide could be the main factor of antibacterial activity (Xie et al., 2011; Sirelkhatim et al., 2015), or binding of ZnO particles on bacterial surface due to the electrostatic forces could be a mechanism (Stoimenov et al., 2002). Therefore, the present study is an attempt to synthesize ZnO and check its potent antimicrobial activity against carbapenem (beta-lactam) resistant strain of *A. baumannii*. The outcome of this study will help to find a suitable alternate to carbapenem, which is currently used to control the infection caused by *A. baumannii*.

## Materials and Methods

**FIGURE 1:**
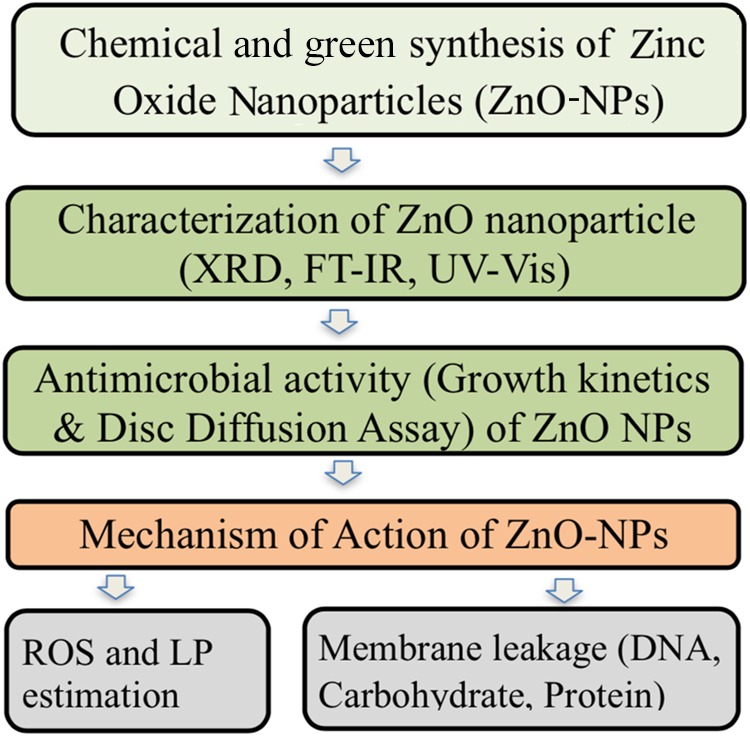
Flowchart representation of different steps used in the present study.

In the present study, ZnO was prepared by chemical and green synthesis methods, and characterized by Fourier transform infrared (FTIR), X-ray diffraction (XRD), and UV-Visible (UV-Vis) spectroscopy. This was followed by anti-microbial activity against three strains of *A. baumannii* using disk diffusion and growth kinetics. The mechanism of action of ZnO was also determined by different biochemical tests. All the methods used in the present study have been outlined in **Figure 1**.

### Bacterial Strain

Two carbapenem-resistant *A. baumannii* (RS-307 and RS-6694) and one carbapenem sensitive (ATCC-19606) strains were used in the present study. These three bacterial strains were available in our laboratory at Central University of Rajasthan, Ajmer. These strains showed the similar pattern in the results hence result of RS-307 is presented here to reduce the repetition.

### Chemical Synthesis of ZnO and Its Characterization

Nanophasic ZnO was successfully synthesized by using acetate precursor-based sol-gel route (Kuberkar et al., 2012; Kansara et al., 2015). Zinc acetate [Zn (CH_3_COO)_2_ × 2H_2_O] was dissolved in 25 mL double distilled water by maintaining 0.4 M solution with continuous stirring at 90°C with 4000 rpm for 30 min. After overlapped processes of polymerization, condensation, and gelation, the gel-based solution was preheated at 150°C for 1 h, to repel out water content from light thick solution. Brown colored powder was then calcined at 250°C for 2 h in high-temperature automated furnace, to remove the organic content from the material. This results in a black fine powder of ZnO. To identify the structural phase present in the ZnO nano-powder, XRD was performed at room temperature using Cu Kα radiation. To understand the size distribution, particle size analyzer was used for dispersed nanoparticles of ZnO. A FTIR spectrum was recorded in the range of 400 to 4000 cm^-1^ for the presently studied ZnO to understand the optical properties of nanoparticles. Water dispersed ZnO nanoparticles (ZnO-NPs) were further analyzed for their optical band gap by performing UV-Vis spectroscopy at room temperature.

### Synthesis of Green ZnO and Antibiotics Capping ZnO

0.2 g of zinc nitrate (0.05 mM) was added to 50 mL water extract of leaf of *Calotropis procera* with constant magnet stirring until complete dissolution as per published protocol (Vijayakumar et al., 2015, 2016). A beta-lactam antibiotics, i.e., ampicillin was tagged to ZnO NPs as per published protocol (Brown et al., 2012; Vijayakumar et al., 2015). The synthesized NPs were characterized using UV-Vis spectroscopy.

### Determination of Anti-bacterial Activity Using Disk Diffusion Assay

Antibacterial assay was performed to study the effect of ZnO on the *A. baumannii* using our published protocol (Tiwari et al., 2016). Disks of chemically synthesized ZnO, conjugated chemically synthesized ZnO, green ZnO, and conjugated green ZnO were used. Disks of distilled water were used as a control. Plates were incubated overnight at 37°C. Antibacterial activity was evaluated by measuring the inhibition-zone diameter (Odebiyi and Sofowora, 1977).

### Growth Kinetics Study of *A. baumannii*

Growth kinetics of *A. baumannii* was determined in the absence or presence of differently prepared ZnO. Bacteria were grown in Luria-Bertani broth in the incubator shaker at 120 rpm, and OD was measured at 605 nm at an interval of 30 min using UV-Vis spectrophotometer as per our published protocol (Tiwari et al., 2017b). The experiment was performed in triplets for control, the presence of chemically synthesized ZnO-NPs, green synthesized ZnO-NPs, ampicillin conjugated ZnO-NPs (chemical or green synthesized), and ampicillin. Relative growth curves of growth kinetics of untreated and treated bacterial culture were prepared for comparison purpose.

### Determination of IC_50_ of ZnO

Inhibitory concentrations of the ZnO-NPs were determined as per published protocol (Gattringer et al., 2002) with some modification. Ten microliters primary culture was mixed with 90 μL LB broth (taken in ELISA plate) and incubated for 2 h at 37°C. Bacterial cultures were treated with different concentrations of ZnO-NPs (1 to 50 mM) and incubated for 5 h at 37°C. This is followed by addition of 5 μL of MTT solution (5 mg/mL) in each well and incubated for 1 h in the dark at 37°C. This step is followed by addition of 100 μL DMSO to each well and incubated again for 2 h at 37°C. After incubation, the OD was monitored at 570 nm and viability rate of each well was determined. Viability percentage is the percentage of the ratio between absorbance of treated wells versus absorbance of control.

### Quantification of Reactive Oxygen Species (ROS)

To estimate the reactive oxidative species produced in the microbial cell, the published protocol was followed (Choi et al., 2006). In brief, a 100 mL bacterial culture was treated with 500 μL of ZnO (final working concentration of 2 mM) and incubated at 37°C in an orbital shaker. After 6 h, bacteria pellet was collected by centrifuging at 10,000*g* for 10 min at 4°C. 2% Nitro Blue Tetrazolium (NBT) solution was added to the pellet, mixed, and incubated for 1 h at room temperature in the dark. This step is followed by centrifugation and supernatant was discarded. Pellet was washed with PBS and centrifuge at 8000*g* for 2 min. Pellet after centrifugation was washed again with methanol and centrifuge at 8000*g* for 2 min. The pellet collected after centrifugation was suspended in 2 M KOH for cell membrane disruption. To this, 50% DMSO solution was added and incubated for 10 min at room temperature to dissolve formazan crystals. It was then centrifuged at 8,000*g* for 2 min. After centrifugation, 100 μL supernatant was transferred to 96 well plates and absorbance was recorded at 620 nm using ELISA reader. Cultures without any treatment were taken as a control, and LB media was taken as blank.

### Quantification of Membrane Lipid Peroxidation

Unstable lipid peroxides cause oxidative stress in microbial cells that decompose to form reactive compounds which lead to cellular damage. Thiobarbituric acid-reactive substances (TBARS) assay is used to detect lipid peroxidation (Joshi et al., 2011; Thombre et al., 2016). In this assay, malondialdehyde forms a complex with thiobarbituric acid, which can be quantified spectrophotometrically. In brief, the 100 mL bacterial culture was treated with 500 μL of chemically synthesized ZnO (final concentration of 2 mM) and incubated at 37°C in an orbital shaker. After 6 h, the culture was centrifuged at 10,000*g* for 10 min at 4°C. The pellet was washed and redispersed in 10%-SDS (500 μL). 20% acetic acid was added to this suspension and incubated for 10 min. 250 μL TBA buffer (0.8% TBA in 2 M NaOH) was added to the solution. This reaction mixture was incubated for 1 h at 95°C and then cooled to 25°C. To remove cell debris, the reaction mixture was again subjected to centrifugation at 5000*g* for 15 min. Absorbance was recorded at 532 nm using ELISA reader. Cultures without any treatment were taken as a control, and LB media was taken as blank.

### Quantification of Membrane Leakage of Reducing Sugars, Proteins, and DNA

The effect of ZnO-NPs on membrane leakage of reducing sugars, proteins, and DNA released from the intracellular cytosol of the cells after treatment with ZnO-NPs was estimated. In the experiment, 100 mL LB broth culture was treated with 500 μL ZnO (2 mM final concentration) and incubated at 37°C in the orbital shaker at 125 rpm. After 24 h, the culture was centrifuged at 10,000*g* for 30 min at 4°C. The obtained supernatant was stored at -20°C. This sample is used for the estimation of reducing sugars, proteins, and DNA contents. Reducing sugar was estimated by Dinitrosalicylic acid assay, which is a colorimetric assay and absorbance was recorded at 540 nm (Miller, 1959). Proteins were estimated by Bradford method and absorbance was recorded at 595 nm (Bradford, 1976). DNA was estimated by absorption spectra at 260 nm (Li et al., 2010; Thombre et al., 2016).

### Transmission Electron Microscopy (TEM)

*Acinetobacter baumannii* was cultured in presence and absence of ZnO. TEM imaging was performed as per published protocol (Tiwari and Moganty, 2014).

### Validation of Cell Viability Using MTT Assay

3-(4,5-dimethylthiazol-2-yl)-2,5-diphenyltetrazolium bromide is a light-sensitive dye, which gives purple color on reduction. It is used in colorimetric assay for assessing cell viability. In this assay, the oxidoreductase enzymes present in metabolically active cells reduce MTT in the cytosol of the cells. The cells with lower metabolic activity reduce very little MTT whereas rapidly dividing cells show higher MTT reduction. Therefore, only living and active cells reduce MTT and those affected cells after treatment unable to reduce MTT. In this assay, the bacterial pellet was suspended in LB media to which 0.5 mg/mL MTT was added. This reaction mixture was incubated for 2 h and then solubilizing buffer was added. Absorbance was recorded at 570 nm (Zhang and Liu, 2002).

### Statistical Analysis

All the experiments were performed in triplicate and data were analyzed by a Student’s *t*-test and a value of *p* < 0.05 was considered significant. Analyzes were performed using Microsoft excel.

## Results

Beta-lactams (carbapenems) are commonly recommended by doctors against *A. baumannii*. The emergence of drug resistance in *A. baumannii* will lead to high mortality and morbidity. Therefore, it is high time to develop alternative molecule against carbapenem resistant *A. baumannii*. In the present study, we have used three strains (RS-307, RS-6694, and ATCC-19606) of *A. baumannii*. These strains showed the similar pattern in result hence result of RS-307 is presented here to reduce the repetition. The RS-307 strain has MIC > 64 μg/mL for the imipenem, i.e., carbapenem resistant strain.

### Synthesis and Characterization of ZnO NPs

**FIGURE 2:**
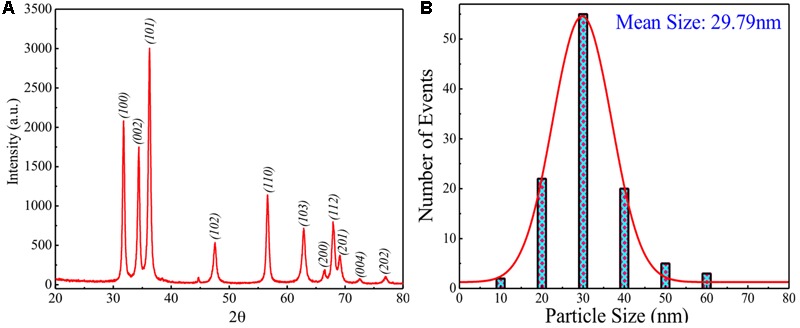
**(A)** XRD pattern of modified sol-gel grown ZnO nanoparticles (ZnO-NPs). **(B)** Particle size distribution with theoretical Gaussian fits for modified sol-gel grown ZnO-NPs.

**Figure 2** shows the XRD pattern recorded for sol-gel grown ZnO powder. It is clearly seen that ZnO, prepared possesses single phasic nature without any detectable impurity within the measurement range. ZnO possesses hexagonal wurtzite unit cell structure. Crystallite size (CS) was calculated using Scherer’s formula: CS = K λ/B cos𝜃, where K is the shape factor, λ is the X-ray wavelength used, B is the peak broadening, and 𝜃 is the angle of incidence. For the present case, K is considered equal to 0.9, by considering the stable spherical shape of the particles. Values of estimated CS are found to be ∼18.72 nm for most intense peak (∼36.26^o^) and ∼17.47 nm by carrying out the average contribution from all the peaks. Difference between these two values suggests the three-dimensional disorder in the nanoparticles.

To understand the size distribution over the presently grown ZnO, particle size analyzer was used at room temperature for dispersed ZnO-NPs. **Figure 2B** shows the particle size distribution for presently studied sample indicating a broad size distribution between the sizes 10–60 nm with the mean size of ∼29.79 nm. This mean size value of particles has been obtained by fitting theoretically the size distribution curve using the Gaussian function. A measurable mismatch between the CS (∼17.47 and ∼18.72 nm) and mean size (∼29.79 nm) can be ascribed to the fact that particle size analyzer has provided result on dispersed ZnO-NPs where the possibility of agglomeration effect exists between the two or more smaller particles to form a larger one.

**FIGURE 3:**
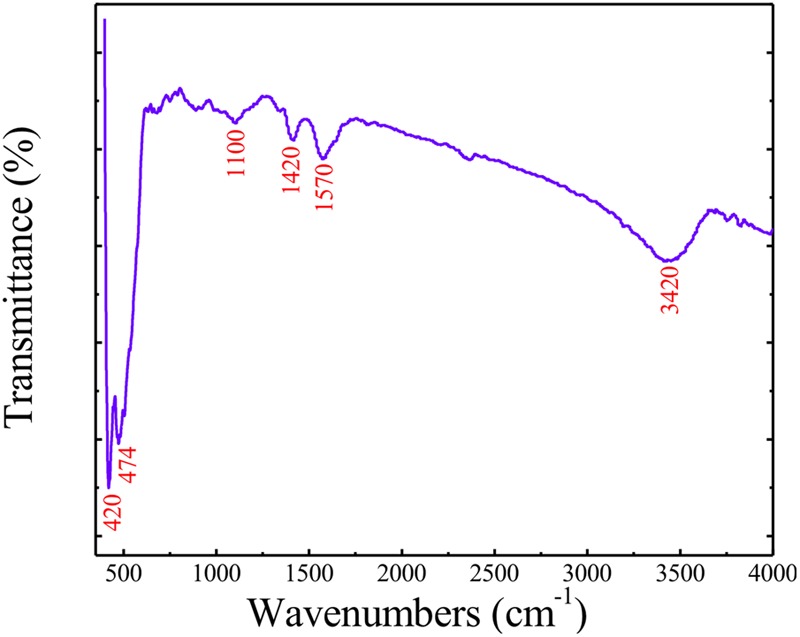
Fourier Transform Infra-Red (FT-IR) spectrum of modified sol-gel grown ZnO-NPs.

To understand the optical properties, purity, and nature of the presently studied ZnO-NPs, FT-IR spectrum was recorded at room temperature, as shown in **Figure 3**. Generally, metal oxides exhibit absorption bands well below 1200 cm^-1^ arising due to interatomic vibrations. As shown in **Figure 3**, the peaks below 500 cm^-1^ (∼420 and 474 cm^-1^) correspond to the Zn-O bonds confirming the formation and purity of ZnO structure (Khana et al., 2015). Next peak ∼1100 cm^-1^ is also due to Zn-O bonds. The peak ∼1420 and 1570 cm^-1^ correspond to COO– (carboxylate group) and C = C bond, respectively (Chithra et al., 2015). Last peak ∼3420 cm^-1^ can be ascribed to the O–H stretching (Chithra et al., 2015).

**FIGURE 4:**
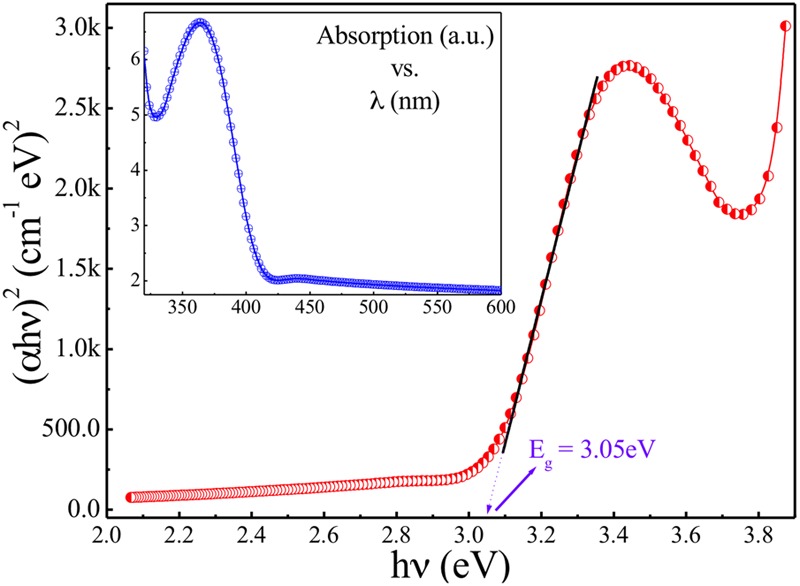
Tauc [(αhν)^2^ vs hν] plot for modified sol-gel grown ZnO-NPs. Inset: absorption spectrum of modified sol-gel grown ZnO-NPs.

Inset of **Figure 4** shows the UV–Vis spectrum carried out for presently studied ZnO-NPs. To estimate the band gap for the presently studied nanoparticles, the Tauc relation can be employed as: αhν = B (hν-E_g_)^γ^, where h is Plank’s constant, E_g_ is band gap, ν is the frequency of incident photon, B is a constant known as band tailing parameter, and γ is the index. If γ = ½ then it is referring to indirect allowed band gap and if γ = 2 then it refers to direct allowed band gap. **Figure 4** shows the Tauc [(αhν)^2^ vs hν] plot for the sample understudy revealing the straight-line fits with the X-axis intercept around 3.05 eV which is the direct band gap of presently studied ZnO-NPs. Similarly, tagging of ampicillin to ZnO and characterization of tagged nanoparticles were done by recording absorption spectra using UV-Vis spectrophotometer.

### Growth Kinetics of *A. baumannii* Under Different Conditions

**FIGURE 5:**
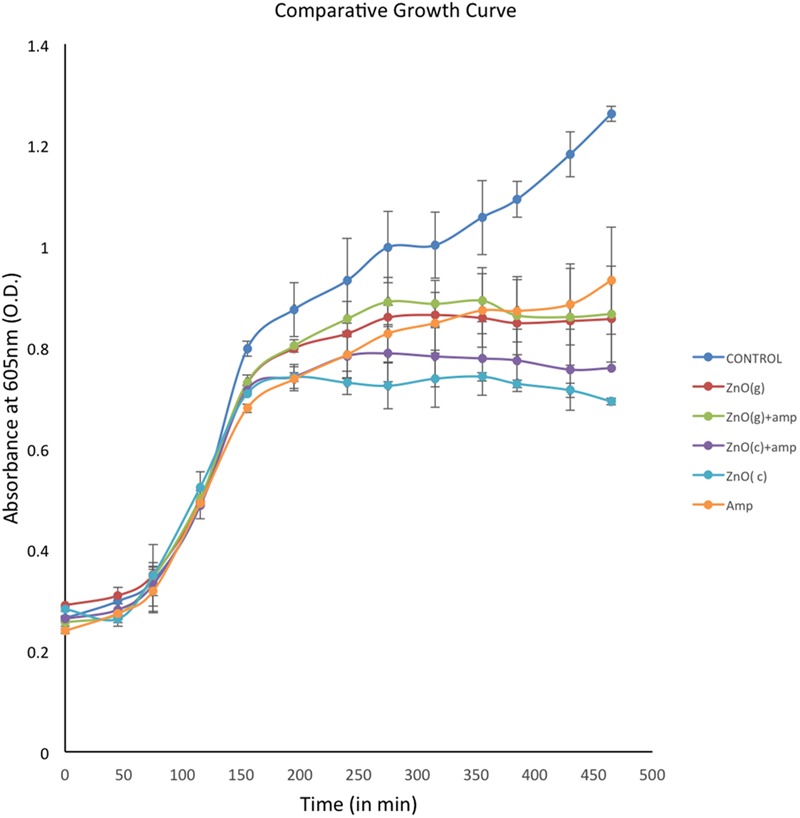
Comparative display of growth curves of *Acinetobacter baumannii* in absence and presence of differentially synthesized ZnO. Experiments were performed in triplicate and data are presented as mean ± SD.

The growth of RS307 strain of *A. baumannii* was analyzed in presence and absence of chemically synthesized ZnO, green synthesized ZnO, ampicillin tagged chemically or green synthesized ZnO, and ampicillin alone. Growth curves of treated bacterial culture experience a decline in comparison to that of the untreated one with time. This suggests that the ZnO, ampicillin, and ampicillin tagged ZnO have activity against the bacterial growth (**Figure 5**). The growth curves in **Figure 5**, shows that treatment of chemically synthesized ZnO inhibits the growth of *A. baumannii* more than the other nanoparticle variants. Green synthesized ZnO nanoparticle and its ampicillin-conjugated variant showed almost similar inhibition on the growth of *A. baumannii*. Ampicillin conjugated chemically synthesized ZnO nanoparticles showed a moderate effect on inhibition of bacterial growth. It is better than green synthesized nanoparticle and ampicillin but not as good as chemically synthesized ZnO nanoparticle (**Figure 5**).

### Antibacterial Activity of ZnO-NPs by Disk Diffusion Assay

**FIGURE 6:**
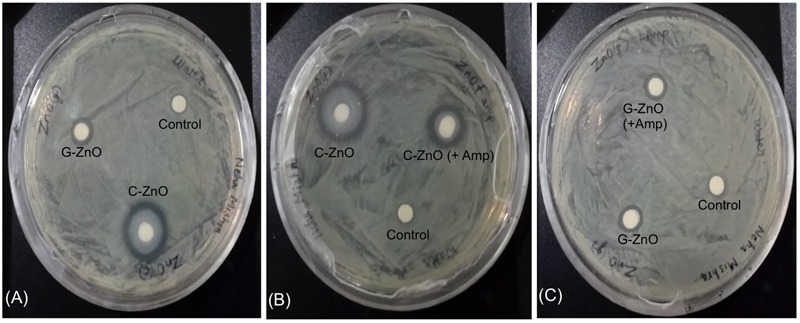
Disk diffusion assay for analyzing the effect of ZnO-NPs on the carbapenem resistant strain of *A. baumannii*. **(A)** Comparative display of chemically synthesized (C-ZnO) and green synthesized (G-ZnO) zinc nanoparticles. **(B)** Comparative display of chemically synthesized (C-ZnO) and ampicillin tagged chemically synthesized (C-ZnO + Amp) zinc nanoparticles. **(C)** Comparative display of green synthesized (G-ZnO) and ampicillin tagged green synthesized (G-ZnO + Amp) zinc nanoparticles.

Disk diffusion assay was performed to analyze inhibition zone of ZnO variants against *A. baumannii*. Antimicrobial activity exhibited by all synthesized nanoparticles, which prevents the growth of bacteria, that can be seen in the form of the clear zone around the disks as seen in **Figure 6**. Here, all of the synthesized nanoparticles showed activity against the RS307 strain of *A. baumannii* but at different levels. The data shows that inhibition zone diameter for chemically synthesized ZnO nanoparticles is maximum as compared to others synthesized nanoparticles. A ring of mucoid growth immediately around disk has also been seen in the C-ZnO treated disks but its reason is unclear. Ampicillin conjugated chemically synthesized ZnO nanoparticle also shows a good inhibition zone. Based on inhibitory effect, the chemically synthesized ZnO nanoparticle has been selected for the study of its mechanism of action study. IC_50_ value of chemically synthesized ZnO NPs was found to be 2 mM for the RS307 strain of *A. baumannii*.

### Effect of ZnO Treatment on ROS Production and Membrane Lipid Peroxidation

**FIGURE 7:**
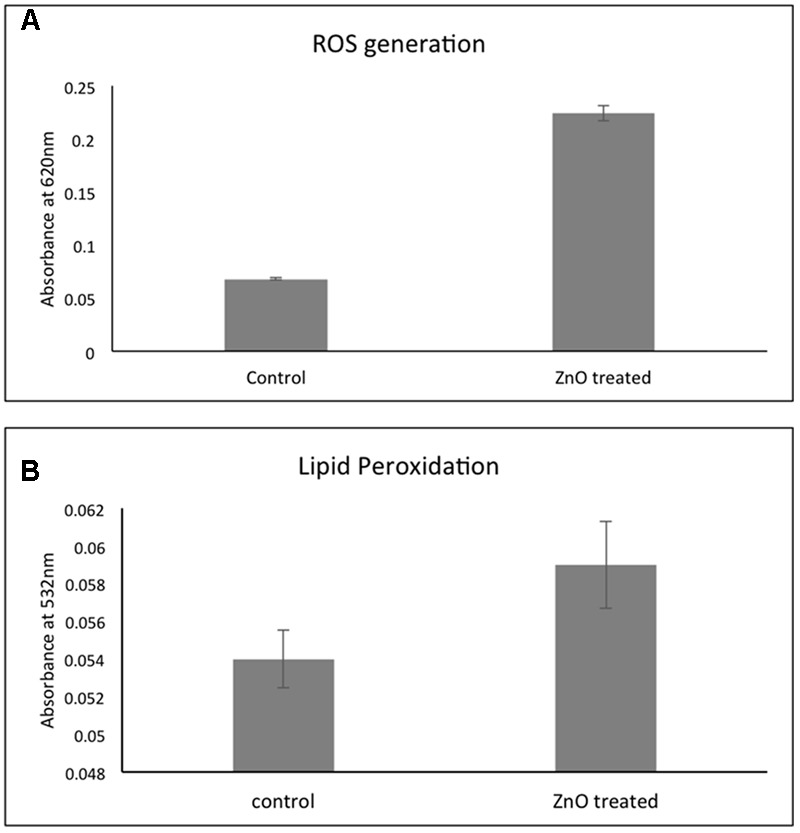
Effect of chemically synthesized ZnO-NPs on the ROS generation **(A)** and lipid peroxidation **(B)** in carbapenem resistant strain of *A. baumannii*. Experiments were performed in triplicate and data are presented as mean ± SD.

Treatment with chemically synthesized ZnO- NPs leads to increase in the formation of reactive oxygen species (ROS) that results into the destruction of the bacterial cells. **Figure 7A** showed that there is a fourfold increase in ROS production in ZnO treated *A. baumannii* as compared to untreated. These elevated ROS leads to have many effects in the bacteria and lipid peroxidation is one of them. The estimation showed that there is a twofold elevation in the lipid peroxidation after treatment with ZnO (**Figure 7B**). This lipid peroxidation effect the bacterial membrane integrity.

### Effect of ZnO on Membrane Leakage of Reducing Sugars, Proteins, and DNA

**FIGURE 8:**
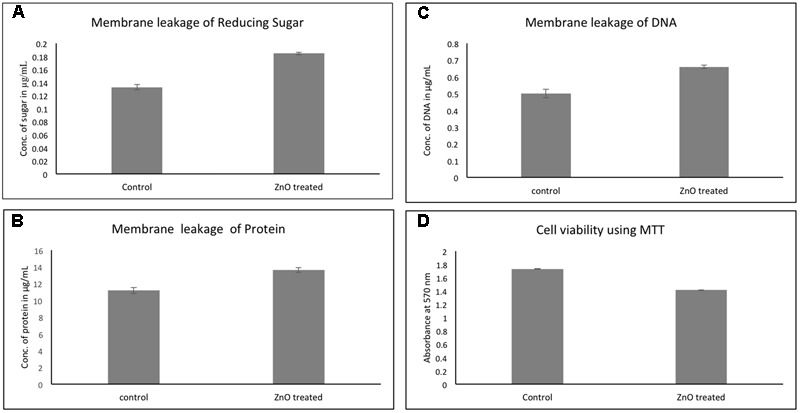
Membrane leakage of carbapenem resistant strain of *A. baumannii* after treatment by chemically synthesized ZnO-NPs. Comparative quantitative display of leakage of sugar **(A)**, protein **(B)**, and DNA **(C)**. Cell viability of RS-307 after treatment with ZnO using MTT assay **(D)**. LB broth media is used as blank and untreated samples are used as control. Experiments were performed in triplicate and data are presented as mean ± SD.

The effect of chemically synthesized ZnO-NPs on membrane leakage of reducing sugars, protein, and DNA was investigated and presented in **Figure 8**. The membrane leakage of reducing sugars was found to be 1.5 times more in ZnO treated bacteria as compared to untreated (**Figure 8A**). Similarly, **Figure 8B** shows that the protein leakage via membrane is around 1.4 times higher after ZnO treatment for 6 h as compared to control (**Figure 8B**). DNA leakage after membrane disruption was 1.3-fold higher after treatment with ZnO (**Figure 8C**). Cultures without any treatment were taken as control.

### Effect of ZnO Treatment on Cellular Viability by Using MTT

In addition to growth kinetics and disk diffusion assay, the cellular viability of *A. baumannii* was assessed using 3-(4,5-Dimethylthiazol-2-yl)-2,5-diphenyltetrazolium bromide (MTT). More metabolically active cells were present in the control (untreated) culture, but after treatment with chemically synthesized ZnO nanoparticle, the number of metabolically active cells in the bacterial culture decreases. Therefore, the lesser number of cells reduces MTT in the treated culture as compared to untreated (control) culture. The cellular viability of *A. baumannii* was found to be decreased by 15% in 2 h of ZnO treatment (**Figure 8D**).

### TEM Results Confirm Membrane Disruption by ZnO-NPs

**FIGURE 9:**
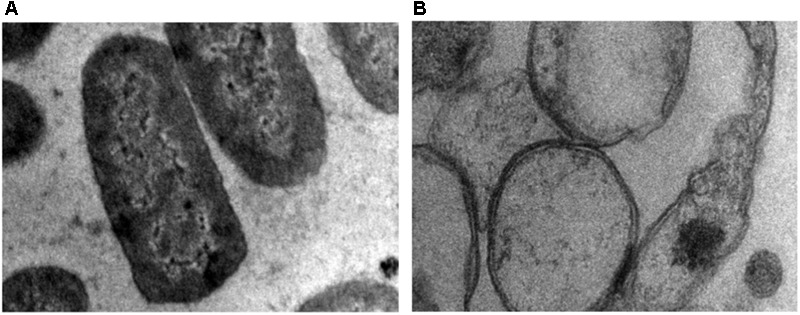
Transmission electron microscopy (TEM) image of *A. baumannii* cultured in the absence **(A)** and presence **(B)** of chemically synthesized ZnO-NPs.

Transmission electron microscopy was performed to study the effect of ZnO on bacterial cell membrane integrity (**Figure 9**). TEM result showed that in the absence of ZnO (control), membrane remains intact (**Figure 9A**) while after ZnO treatment membrane rupture takes place that releases cell contents. After treatment, cells were empty, and cytoplasm is diffused out of cell membranes due to membrane rupture (**Figure 9B**). This suggests that ZnO kill bacteria via disrupting cell membrane. TEM results showed good correlation with our biochemical assays like estimation of ROS, lipid peroxidation, and release of cell contents.

## Discussion

*Acinetobacter baumannii* is a pathogenic Gram-negative bacterium, which is one of the ESKAPE pathogens with multi-drug resistance. The emergence of resistance in *A. baumannii* causes high mortality and morbidity. *Acinetobacter* has developed an ability to accumulate diverse resistance mechanisms. The rise in the antibiotic resistance and emergence of antibiotic-resistant superbugs, stressing the need for strategies to developing new antimicrobial. Therefore, there is an urgent call for the discovery of new drugs or new target of the old molecules that are capable of enhancing the efficacy of current therapy. Therefore, there is a high time to develop antibiotic alternative therapeutics against carbapenem resistant *A. baumannii*. There are different approaches that can be employed which includes herbal-based (Tiwari et al., 2015b, 2017a; Roy et al., 2018), *in silico* based (Tiwari et al., 2018a,b; Verma et al., 2018), nanomaterial-based (Tiwari et al., 2015a, 2017b; Wan et al., 2016), and combination therapy (Saballs et al., 2006; Song et al., 2008; Jain et al., 2009; Aydemir et al., 2013; Garnacho-Montero et al., 2013; Hiraki et al., 2013; Habash et al., 2014). These approaches are tried recently against *A. baumannii* and some of them shown very promising results.

Recently, ZnO NPs have shown antimicrobial activity on skin-specific bacteria (Aditya et al., 2018), *Streptococcus mutans, Streptococcus pyogenes, Vibrio cholerae, Shigella flexneri*, and *Salmonella typhi* (Soren et al., 2018). It has also shown antimicrobial activity against methicillin resistant *Staphylococcus aureus* (Kadiyala et al., 2018). Antimicrobial activity of metallic nanoparticle like PVP-capped AgNPs (Tiwari et al., 2014, 2015a, 2017b) and citrate-capped AgNPs (Wan et al., 2016) were studied in *A. baumannii*. One study has been done so far where antibiotic-coated ZnO NPs showed anti-microbial activity against *A. baumannii* (Ghasemi and Jalal, 2016), but no study has been done so against the carbapenem-resistant strain of *A. baumannii*. Interaction of metallic NPs with different cell models and their cellular effect have been reviewed recently (Zhang et al., 2016) and showed the involvement of ROS during the interaction of NPs with different cell lines.

In the present study, the carbapenem resistant strain of *A. baumannii* was used to check the antimicrobial activity of synthesized ZnO compounds. ZnO nanoparticles were synthesized chemically and by the green method. Synthesized ZnO-NPs was characterized by XRD, FTIR, and UV-Vis spectroscopy. All of these nanoparticles were then tested for their antimicrobial activity that indicated that chemically synthesized ZnO nanoparticle shows the good inhibition in comparison to other synthesized nanoparticle variants. Furthermore, mechanism of effect of ZnO nanoparticles on *A. baumannii* were assessed on different parameters like ROS generation; lipid peroxidation; membrane leakage of reducing sugars, proteins, DNA, and cell viability. TEM result also confirms the membrane disruption after ZnO-NPs treatment. Based on all the results, the proposed mechanism of action of ZnO involves the production of ROS, which elevates membrane lipid peroxidation that causes membrane leakage of reducing sugars, proteins, DNA, and reduces cell viability.

## Conclusion and Future Perspectives

Therefore, it can be concluded from the present study that chemically synthesized ZnO-NP can be developed as an alternative to carbapenem (beta-lactam), that inhibit the growth of carbapenem resistant *A. baumannii* by producing ROS and causing membrane damage. Therefore, chemically synthesized ZnO nanoparticles can be more favorable future hope as an alternative drug to carbapenem against this carbapenem-resistant strain of *A. baumannii*.

Similarly, pathogenicity of *A. baumannii* is influenced by its ability to survive in the human pulmonary cells. Therefore, it is also important to study effect of the ZnO in the interaction of *A. baumannii* with the human pulmonary host cell. The pulmonary cell-targeted delivery of ZnO in animal model need to be further validated to make it as a suitable drug against *A. baumannii*. Cell line and animal-based studies are also critical to have an improved mechanistic knowledge under *in vivo* setup. Detailed proteomic studies of *A. baumannii* in the presence of ZnO, is also required to identify the proteins involved in the mechanism of action of this molecule. Cytotoxicity of chemically synthesized ZnO-NP can be tested to determine the effective non-cytotoxic dose of the ZnO for cell line and human model.

## Author Contributions

VT conceived and designed the experiments, wrote the manuscript, and proofread of final version. NM, MT, KG, and VT performed the experiments. VT, PS, and NS analyzed the data.

## Conflict of Interest Statement

The authors declare that the research was conducted in the absence of any commercial or financial relationships that could be construed as a potential conflict of interest.
